# What predicts mental health literacy among school teachers?

**DOI:** 10.4314/gmj.v55i2.7

**Published:** 2021-06

**Authors:** Vidya Prabhu, Lena Ashok, Veena G Kamath, Varalakshmi C Sekaran, Asha Kamath, Sebastian Padickaparambil, Asha P Hegde, Virupaksha Devaramane

**Affiliations:** 1 Melaka Manipal Medical College (Manipal Campus), MAHE, Manipal - Department of Community Medicine, Udupi, India; 2 Department of Global Health, Prasanna School of Public Health - MSW Program, MAHE, Manipal, Manipal, Karnataka 576104, India; 3 Kasturba Medical College Manipal - Department of Community Medicine, Manipal, Karnataka, India; 4 Melaka Manipal Medical College (Manipal Campus) - Department of Community Medicine MAHE, Manipal, Karnataka 576104, India; 5 Prasanna School of Public Health, MAHE, Manipal - Department of Data Science, Udupi, Karnataka, India; 6 Manipal College of Health Professions (MCHP), MAHE - Department of Clinical Psychology, Udupi, Karnataka, India; 7 Melaka Manipal Medical (Manipal Campus), MAHE, Manipal - Department of Paediatrics, Udupi, Karnataka, India; 8 Dr. AV Baliga Memorial Hospital - Department of Psychiatry, Udupi, Karnataka, India

**Keywords:** Mental health literacy, teachers, adolescent, predictors

## Abstract

**Objectives:**

The present study aimed at assessing high school teachers' mental health literacy (MHL) and predictors related to study outcomes.

**Design:**

Cross-sectional study

**Methods:**

We employed 460 high school teachers who engaged with adolescents for at least six hours per week with a minimum of five years of teaching experience in southern India. Semi-structured questionnaires were used to assess their MHL. Descriptive analysis and backward logistic regression analysis were performed. A p-value < 0.05 was set as significant.

**Results:**

Teachers' MHL on depression was less than desirable; however, they identified 288 (62.6%) adolescents with mental health problems during their career, and 172(59.72%) were referred to mental health professionals. On logistic regression analysis, teachers' educational status, their marital status, teaching a class with an average strength of 31-60 students per class, previous mental health training and having self-efficacy concerning seeking information on mental health, perceived ability to spread awareness and to provide referrals were found to predict MHL among teachers.

**Conclusion:**

Sociodemographic factors including teachers' educational status, average class strength and having had previous mental health training were predictors for MHL among high school teachers. Establishing training programs and referral networks may be key in early intervention among adolescents.

**Funding:**

This work was funded by the Indian Council of Medical Research (ICMR) (No. Adhoc/2015/85/HSR)

## Introduction

Mental health is an integral part of human beings' overall health at every phase of life, from childhood to adulthood. According to the World Health Organization, universally, 10%-20% of children and adolescents have mental health problems,^1^ with every one among four children suffering from some form of mental health problem.

Mental health problems are recognised as a major contributor to the overall burden of disease among the young, and the number of cases is rising.^2^,^3^

The association between mental health problems and suicides have been established in the literature and adds to the growing concern that it is now counted as the third leading cause of death among adolescents.^4^

Several studies have been conducted across the globe to assess the burden of mental health problems among children and adolescents. A study in the USA reported the lifetime prevalence of mental health problems among adolescents and found anxiety to be the most common condition. The prevalence rate of anxiety disorders was 32%, mood disorders at 14%, behavioural disorders 19% and substance use disorder at 11%.^5^ A survey from Australia reported a prevalence of 13.9% mental health problems among 4-17-year-olds.^6^

There has been an increasing interest in identifying mental health problems among children and adolescents in India. Studies conducted in India have shown varying degrees of mental health problems among Indian adolescents, including depression, anxiety, schizophrenia, and substance use disorders. Bhola and Kapur,^7^ in their review, identified 23 school-based studies with prevalence ranging from 3.2% to 36.5%. Srinath et al.,^8^, in their study initiated by the ICMR at two centers in India among children and adolescents aged between 0 years -16 years, revealed a prevalence rate of 12.5%. According to a recent review, the prevalence of child and adolescent mental health problems in the community setting was 6.4% and 23.3% in the school setting.^9^ Hasumi T et al., 10 found that depression was prevalent among Indian adolescents and increased with age. Increasing stress among adolescents has been associated with anxiety, withdrawal, and depression as well.^11^ Sharma and Sharma,^12^ also found that substance abuse such as smoking also increased anxiety and stress levels.

Mental health during adolescence is important because half of the people who develop mental health problems at a later time in life do have their first episode before 14 years of age.^13^ Early identification and treatment of mental health problems during adolescence can result in a better prognosis and functional outcome in adulthood. Unrecognised and untreated, mental health problems could severely influence children's development, educational attainments, and potential to live fulfilling and productive lives. With the increasing thrust towards attaining the Sustainable Development Goal four of providing quality education, adolescents spend about eight hours interacting with their peers and teachers in the school setting. Hence, the school is an important environment where teachers may play a neutral role in identifying and facilitating referrals for adolescents' mental health needs.^14^ Studies have reported that teachers may act as key resources in promoting mental health among adolescents because a link between educational attainment and mental well-being has been established in the literature.

Teachers may be able to identify changes in their wards' behaviour during their interactions before others noticing them, provided they have adequate knowledge of mental health problems themselves.^14^,^15^

MHL assessment among teaches across the globe has reported poor mental health literacy.^16^,^17^ In India, a few studies have explored MHL and have reported inadequate knowledge and stigma against seeking help for mental health problems.^4^,^7^,^8^,^9^,^15^ To the best of our knowledge, there is a shortage of data on teachers' perceived self-efficacy to facilitate services for adolescent mental health needs, their MHL, referral practices for adolescents seeking mental health services and predictors for these outcomes. This study attempts to bridge the gap.

## Methods

A cross-sectional approach was undertaken to conduct the study among schools in Udupi taluk. A stratified cluster random sampling technique was adopted to stratify schools into government, aided, and private schools. From each category, the schools were selected using a simple random sampling technique to achieve the required teacher population under each category calculated. The inclusion criteria for high-school teachers should have a minimum of five years of teaching experience and engage with adolescents for at least six hours per week.

Ethical approval to conduct the study was obtained from the Institutional Ethics Committee of KMC and KH, MAHE, Manipal with no. 447/2017. Permission was sought from the Deputy Director of Public Instructions (DDPI), concerned Block Education Officers, and school authorities.

Considering 30% MHL among the teachers derived from a pilot study with the absolute precision of 6%, 95% confidence interval (CI) and a design effect of 1.5, accounting for 20% attrition, a minimum of 420 teachers were required. In all, 460 teachers participated and provided data. Before data collection, written informed consent was obtained from the participants.

A semi-structured validated and pretested tool was used to collect data. It was developed based on the “Australian National Mental Health Literacy and Stigma Youth Survey”,^18^ with permission from the authors. The questionnaire was developed in English and translated into the local language, Kannada. Back-translation into English was performed to check the consistency. Content validation of the tool was done by professionals from the field of Psychiatry and Clinical Psychology. The questionnaire included the following domains: socio-demographic characteristics of participants, case vignettes describing depression, self-efficacy of participants on identifying and providing referral for mental health needs of adolescents, and practice-related questions on prior identification and referral over their career.

The vignettes and some questions were modified based on the suggestions provided during validation to fit the Indian context. The description of symptoms in the depression vignette met that of the International Classification of Diseases (ICD)-Eleventh Edition diagnostic criteria. On the vignette, contrary to questionnaires that provide a drop-down list of options to participants, no options were provided to assess the participants' actual knowledge regarding the symptoms presented. The vignette described an adolescent who exhibited behavioural changes over three weeks, including feeling sad, sleeplessness, weight loss, and not expressing interest in activities of interest to the adolescent. Data were analysed using SPSS version 15. Descriptive data were reported in percentages and frequencies. Backward logistic regression was used to identify predictors.

## Results

The socio-demographic characteristics, teaching and work schedule of teachers are depicted in Table 1. Of the total 460 participants, 173 (37.6%) were in the age group of 36-45 years and were predominantly female 322 (70%) and 289 (62.8%) of them were postgraduates. About 215 (46.7%) had five to nine years of experience and taught in private schools 182 (39.6%). More than half of the participants taught anywhere between 16-25 hours per week and 240 (52.2%) with the Kannada language as the medium of instruction 260 (56.5%). Most of the participants, 272 (59.1%) reported teaching an average class strength of 31-60 students per class.

**Table 1:** Socio-demographic profile of study participants

Variables		n (%)
**Age in years**		
	26-35	118 (25.7)
	36-45	173 (37.6)
	46 and above	169 (36.7)
**Gender**		
	Male	138 (30)
	Female	322 (70)
**Income (Indian Rupee)**		
	<15000	34 (7.4)
	15001-30000	123 (26.7)
	30001-45000	93 (20.2)
	>45000	210 (45.7)
**Religion**		
	Hindu	385 (83.7)
	Christian	67 (14.6)
	Muslim	6 (1.3)
	Others	2 (0.4)
**Level of education**		
	Diploma and others	18 (3.9)
	Graduation	153 (33.3)
	Post-graduation	289 (62.8)
**Marital status**		
	Single	31 (6.7)
	Married	424 (92.2)
	Widowed	5 (1.1)
**Type of family**		
	Single	17 (3.7)
	Nuclear	286 (62.2)
	Joint	152 (33)
	Extended	5 (1.1)
**Teaching experience in years**		
	5-14	215 (46.7)
	15-24	170 (37)
	24 and above	75(16.3)
**Status of work**		
	Permanent	364 (79.1)
	Temporary	96 (20.9)
**Type of school**		
	Government	151 (32.8)
	Private	182 (39.6)
	Aided	127 (27.6)
**Hours of teaching per week in hours**		
	6-15	88 (19.1)
	16-25	240 (52.2)
	26 and above	132 (28.7)
**Medium of instruction**		
	Kannada	260 (56.5)
	English	175 (38)
	Mixed	25 (5.4)
**Average class strength**		
	<=30	156 (33.9)
	31-60	272 (59.1)
	>61	32 (7)
**Type of syllabus**		
	State	366 (79.6)
	CBSE	71 (15.4)
	ICSE	23 (5)

Participants' perceived self-efficacy on identifying and providing referral for mental health needs of adolescents were assessed. Individual items on their ability to spread awareness 353 (76.7%), ability to identify behavioural problems among their wards 432 (93.9%), providing referral 382 (83%), ability to assist parents in seeking help for observed behavioural changes among their children 417 (90.7%), and their self-efficacy on reducing school dropouts or sickness absenteeism concerning behavioural problems among adolescents 386 (83.9%) were assessed. Overall, teachers reported high levels of perceived selfefficacy to spread awareness 353 (76.7%) and the ability to provide referral 382 (83%). Of the 460 participants, 296 (64.4%) expressed willingness to work on school duties with people having mental problems.

The MHL concerning depression was low, with 68(14.8%) identifying depression from the given vignette appropriately.

About practices related to MHL, a greater proportion stated that they had undergone some form of training regarding mental health 95 (20.7%).

More than half of the participants stated that they had accessed information regarding mental health on their own interest 280(60.9%). The majority of the participants had identified adolescents with mental health needs 288 (62.6%), and among them, 172 (59.72%) had referred them to mental health professionals (Table 2).

**Table 2:** Teachers' practices related to mental health literacy (n=460)

Characteristics	Yes n(%)	No n(%)
**Undergone training regarding mental health**	95(20.7)	365(79.3)
**Identified adolescents with mental health problems**	288(62.6)	172(37.4)
**Referred adolescents with mental health problems (n=288)**	172(59.72)	116(40.28)
**Ever looked for information regarding mental health**	280(60.9)	180(39.1)
		

On assessing for predictors, factors that were protective towards identifying any mental health condition among adolescents during their career emerged. These factors included the marital status of the participant, i.e., being single or married (AOR =0.05, 95% CI 0.005-0.62; p=0.019) and (AOR=0.06, 95% CI 0.006-0.61; p=0.017)] in comparison with widowed participants, having previously sought information on mental health (AOR= 0.32, 95% CI 0.20-0.50; p=0.0001), their self-efficacy in relation to perceived ability to spread awareness on mental health (AOR=0.42, 95% CI 0.235-0.743; p=0.003) and perceived ability to provide referral (AOR=0.50, 95% CI 0.273-0.924; p=0.027) if the need arose.

On assessing for predictors regarding the identification of depression using the vignette, having had postgraduate education predicted a higher odds of identification as opposed to graduate education (AOR =4.37,95% CI 1.97-9.72; p=0.0001) while a smaller class strength of 31 to 60 students played a protective role (AOR= 0.19, 95% CI 0.04-0.95; p=0.042) in comparison with class strength above 60 students which hampered identification.

Factors that predicted participants' referral of adolescents to mental health professionals for their mental health needs including prior training on mental health in comparison with untrained participants (AOR= 0.47, 95% CI 0.28-0.79; p=0.004), having previously sought information on mental health (AOR= 0.48, 95% CI 0.28-0.79; p=0.003) and their perceived ability to provide referral (AOR= 0.34, 955 CI 0.17-0.70; p=0.004) (Table 3).

**Table 3:** Predictors identified for mental health literacy

Predictors for identification of mental health problems among students			
Variable		AOR(CI)	p-value
**Marital status**			
	Single	0.05(0.0050.62))	0.019
	Married	0.06(0.006-0.61)	0.017
	Widowed	1	
**Seek information on mental health**			
	Yes	0.32 (0.20-0.50)	0.0001
	No	1	
**Able to spread awareness**			
	Yes	0.42(0.2350.743)	0.003
	No	1	
**Able to provide referral**			
	Yes	0.50(0.2730.924)	0.027
	No	1	
**Predictors for mental health literacy on depression based on the vignette**			
**Variable**		AOR(CI)	p-value
**Level of education**	Diploma	8.23(0.9968.42)	0.05
	Graduation	4.37(1.97-9.72)	0.0001
	Post-graduation	1	
**Average class strength**	<=30	0.2 (0.04-1.34)	0.05
	31-60	0.19(0.04-0.95)	0.042
	>60	1	
**Predictors for providing a referral to a mental health professional**			
**Variable**		AOR (CI)	p-value
**Underwent training**			
	Yes	0.47 (0.28-0.79)	0.004
	No	1	
**Seeking information on mental health**			
	Yes	0.48 (0.28-0.79)	0.003
	No	1	
**Able to provide referral**			
	Yes	0.34 (0.17-0.70)	0.004
	No	1	

### Limitations

The present study is a cross-sectional study. Therefore, we are unable to make causal inferences. Identification of mental health problems was based on self-report and may be subject to recall bias or social desirability bias.

## Discussion

The present study aimed at assessing high school teachers' MHL and predictors related to study outcomes. Depression presents as a common mental health problem among adolescents and studies have emphasized the need to explore MHL among teachers.

Perceived self-efficacy of teachers in relation to identifying mental problems, perceived ability to spread awareness on mental health, and to provide referrals were reportedly high; however, the actual identification of depression using the vignette was low at 68(14.8%). Our findings are in line with results from a study conducted in Nigeria by Aluh DO et al., who assessed MHL concerning depression among high school teachers and found that it was 16.3%.^17^

In the Indian context, studies have focused on knowledge regarding etiology, symptoms, and attitudes and beliefs surrounding mental health and management. However, these studies seldom included teachers. In the state of Gujarat, Parikh et al.,^15^ assessed teachers' knowledge of adolescent mental health problems and reported that 76% of teachers had less than desirable scores and high rates of stigma. However, our participants expressed willingness to work closely on school duties with people with mental health problems, indicating a lower level of stigma in comparison. Mendonsa RD,^4^ conducted a study at the adjacent district of Mangalore on the knowledge and beliefs among elementary school teachers in relation to mental health and again reported poor knowledge (mean score of 4.5±2.23). However, predictors in relation to identification and referral have not been documented.

Studies that probed for predictors of MHL among teachers are limited; however, predictors related to MHL in the general population have been conducted in several settings globally^19^-^22^ with a higher socioeconomic status emerging as a significant predictor. However, this was not a significant predictor in our study. The marital status of the participant and self-efficacy in relation to seeking mental health information perceived ability to spread awareness and provide referral predicted identification of mental health problems among their wards were significant in this population.

The current study also revealed that referral for mental health needs of adolescents over the participants' career was higher when they had previously undergone mental health training, when they had accessed mental health information of their interest and when they reported perceived self-efficacy to provide the referral. Similar predictors were not found among teachers in literature. However, a systematic review and meta-analysis of mental health first aid training conducted by AJ Morgan et al.,^23^ among the general population, including teachers reported that training led to improvements in identifying mental health problems and confidence in helping a person with a mental health problem also improved.

## Conclusion

Sociodemographic factors including high school teachers' educational status, average class strength and having had previous mental health training were predictors for MHL. The mental health literacy of the teachers is low.

Based on our findings, training programs on adolescent mental health targeting teachers is the need of the hour. Participants' readiness to work with adolescents who may need to access mental health services was high as evidenced by perceived self-efficacy implying lower stigma. Teachers can be effective change agents if provided adequate training and can act as an important liaison between adolescents who need mental health services and mental health professionals.
